# University Student Attitudes Toward Persons With a History of Adult Sexual Violence and Their Correlates With Policy, Treatment, and Management

**DOI:** 10.1177/08862605221109892

**Published:** 2022-07-22

**Authors:** Gabriela Corăbian, Neil R. Hogan, Mark E. Olver

**Affiliations:** 1 Alberta Hospital Edmonton, Canada; 2 Integrated Threat and Risk Assessment Centre, Edmonton, AB, Canada; 3 University of Saskatchewan, Saskatoon, Canada

**Keywords:** sexual violence, attitudes, public policy, reintegration, desistance

## Abstract

Empirical evidence suggests that postsecondary students are disproportionately impacted by sexual violence. Further evidence suggests that most persons convicted of crimes involving sexual violence return to the community, and that social factors, including public policy and community members’ attitudes and perceptions, are key determinants of successful reintegration among these persons. Taken together, these observations suggest that students’ perspectives should be considered in discourse related to reintegration. The current study aimed to assess the attitudes of a university undergraduate sample toward men convicted of adult sexual violence as well as their level of support for various community-based policies to manage this population upon release from custody. Participants (*N* = 333) completed a survey battery comprising measures of three dimensions of attitudes toward persons who have sexually offended, perceptions of recidivism and treatment response, and support for various incapacitation/control (IC) and rehabilitative/reintegration (RR)-based policies. Results varied depending on the dimension of attitudes measured, with the affective component appearing to be the most markedly negative. Participants endorsed a mixture of IC and RR policies, with attitudinal measures predicting policy endorsement controlling for relevant demographic variables. The results provide a framework for future research surveying a more representative sample of the Canadian public, while also providing useful information for policymakers relying on community support to reduce sexual offending.

Sexual violence is endemic to postsecondary settings. While precision in estimating the scope of the problem is limited by variability in definitions, time scales, and approaches to collecting data, there is little doubt that students are at elevated risk of sexual victimization (Fedina et al., 2018). Recent Canadian survey data (Burczycka, 2020) obtained from a broadly representative sample of postsecondary students indicated that approximately 71% of respondents reported either witnessing or directly experiencing unwanted sexual behavior in 2019. During the same period, 11% of female respondents and 4% of male respondents reported having been the victim of a sexual assault in the postsecondary setting. Expanding the period in question to entire university careers, the reported rates of sexual assault for these two groups rose to 15% and 5%, respectively. Given that they are disproportionately impacted, it stands to reason that postsecondary students should be invited to contribute to the discourse with respect to societal and policy responses to sexual violence.

Meaningful responses to sexual violence are necessarily multifaceted and involve many parties. One critical area of focus involves responses to justice-involved persons who have previously perpetrated sexual violence, because most of these individuals will reside in the community during (i.e., while undergoing community supervision) or after serving their sentences. Successful desistance from offending and community reintegration are interrelated phenomena, and factors such as social support, housing, and employment are known to predict recidivism (Hanson & Harris, 1998). Given that these types of needs can typically only be met with the engagement of the community, community members’ attitudes and public policies can exert significant influences on sexual recidivism (Willis et al., 2010). Featuring a university undergraduate sample, the current study aimed to assess attitudes toward persons who have been convicted of a contact sexual offense against a non-consenting adult, and their correlates with respect to various community-based policies to manage risk upon return to the community.

## Perceptions of, and Attitudes Toward, Persons with Sexual Offense Histories

Objective and informed responses to sexual violence can be difficult, given stigma and misinformation. Members of the public, including postsecondary students, tend to overestimate formally detected sexual offending and recidivism rates, and to endorse other negative and erroneous stereotypes about persons with sexual offense histories (e.g., Brown et al., 2008; Fortney et al., 2007; Levenson et al., 2007; Olver & Barlow, 2010). Criminal justice professionals are also susceptible to bias, as evidenced by an observed tendency to skew formal risk appraisals upward when evaluating people with sexual offense histories (Orton et al., 2021). In addition, surveyed professionals, people with sexual offense histories, and the public have opined that media portrayals impede community reintegration by limiting opportunities for housing, employment, and relationships (Corăbian & Hogan, 2012). Thus, the study of public perceptions and attitudes toward this target population is essential to the pursuit of a fuller understanding of community reintegration and desistance.

## Public Policy and Professional Practice Measures for Managing Sexual Violence

People who commit sex offenses are diverse in their criminogenic needs, risk profiles, and offending patterns, and policies designed to manage them are necessarily diverse. Some policies are specific to postsecondary settings, while others are more generally relevant to the broader community. Notably, campus-based initiatives to combat sexual violence are wide ranging (McMahon et al., 2019; Richards, 2019), focusing on domains such as prevention programming; bystander intervention; reporting, investigatory, and disciplinary procedures; support for persons who have been victimized; and links between campus responses and typical criminal justice processes. While these campus-focused initiatives warrant study in their own right (McMahon et al., 2019), the current study focuses on responses to persons who are known to have already perpetrated sexual violence, which tend to be less campus specific. It is important to note that although campus sexual violence is sometimes treated and studied as a phenomenon that is conceptually distinct from other forms of sexual violence, perpetrators of sexual violence against university students are not exclusively members of campus communities themselves, and sexual violence experienced by university students does not always occur on university campuses. The previously cited study conducted by Burczycka (2020), for instance, in which postsecondary students reported having experienced sexual violence at high rates in “the postsecondary setting” (p. 6), included in this category incidents occurring: while traveling to school (e.g., on public transit), while completing external work placements, or “online where some or all of the people involved were students” (p. 5). Furthermore, some of the most egregious sexual violence perpetrated against university students on campuses, including incidents culminating in homicide, have involved persons without any apparent connection to the campus community (e.g., Rolling v. State of Florida, 1997). Thus, when seeking to address sexual violence with the interests and well-being of university students in mind, a broad perspective which includes a focus on persons convicted of sexual offences is warranted.

A number of policies and practices are employed in Canada and the United States to manage persons who have been convicted of sexual offenses. One approach is registration and community notification, whereby individuals’ information is collected in a database that may be made available to law enforcement or the public in various forms (Cucolo & Perlin, 2013; Murphy et al., 2009). Second, residence restrictions constrain where individuals may go about their daily activities and may include prohibiting them from attending locations where potential victims may frequent (e.g., university campuses). Third, restrictive sentencing and supervision policies are common, examples of which include mandatory minimum sentences; preventative civil commitment under Sexual Violent Predator laws (Levenson, 2003); and long or indeterminate detention or supervision periods under Canadian Dangerous Offender, Long Term Offender, or Criminal Code Section 810 Recognizance Orders (Lussier et al., 2014; Public Safety Canada, 2015). A final set of policies and practices pertain to treatment/rehabilitation interventions, increased structure and supervision, and community support to prevent sexual recidivism and aid reintegration. Examples include electronic monitoring policies, including Global Positioning System (GPS) tracking; sexual-offense-specific psychological/behavioral treatment programs and libido-reducing pharmacological treatment (Kutcher, 2010); community reintegration projects, such as Circles of Support and Accountability (COSA; Petrunik, 2002); and comprehensive and integrated correctional programming, based on principles known as risk (match treatment intensity to risk level), need (target dynamic risk factors for services), and responsivity (individually tailor treatment services using cognitive behavioral methods), or risk-need-responsivity (RNR) (Andrews & Bonta, 2010).

Readers should note that while each of the aforementioned policy types have been applied to persons with sexual offense histories in some form, they have not all received similar support for their effectiveness. For instance, while a recent meta-analytic review found support for sexual-offense-specific treatment programs (Gannon et al., 2019), considerable research to date has failed to provide supportive evidence for the use of registration and notification legislation to prevent sexual recidivism (e.g., Tewksbury & Jennings, 2010; Vasquez et al., 2008). Similarly, whereas interventions guided by the RNR model appear effective for reducing sexual recidivism (Hanson et al., 2009), most research investigating the impacts of residence restrictions suggests that this approach is not so (Galeste et al., 2012; Levenson, 2011). Thus, in terms of their effectiveness for reducing sexual violence, not all policies are created equal.

## Current Study Rationale and Research Objectives

Extant evidence suggests that sentiments toward persons convicted of sexual offenses are generally very negative around the world and among different public sectors including health, university, and related settings. Furthermore, Canadian government policy developments within the previous decade have adopted a punitive, but not necessarily evidence-based approach (e.g., Safe Streets and Communities Act, 2012; Tougher Penalties for Child Predators Act, 2015), reflecting political and perceived public sentiment toward people who break the law in general, and commit sex offenses in particular. It is unclear, however, how Canadian attitudes and perceptions in general, and those of a group of people closely impacted by sexual assault (i.e., university students) in particular, relate to support for particular policies. In our view, it is important to collect and analyze data about psychological phenomena not just at a broad population level, but also at the subgroup level, because patterns observed among subgroups do not necessarily reflect global patterns (i.e., Simpson’s Paradox; see Kievit et al., 2013). Furthermore, to our knowledge, research has yet to examine how public sentiment toward a group that commit crimes, that provoke the level of fear and ire in the way that sex offenses do (and which heavily impact university campus communities), is related to structured operationalizations of prevention and management policies as developed for the purposes of the present study.

Focusing on a Canadian university undergraduate sample, this study examined the attitudes and perceptions relating to persons convicted of sexual offenses against adults, and associations with potential policies at a broader societal level to reduce sexual violence. The context of the study sample is significant, given the aforementioned concerns regarding sexual violence experienced by postsecondary students, and also given that female students (who comprise the bulk of the study sample) are about 2.5 times more likely to be sexually assaulted in some form than their male counterparts (Burczycka, 2020; Westat, 2020).

The current study had four primary research objectives. First, we sought to examine Canadian postsecondary students’ attitudes toward persons convicted of sexual offenses (specifically sexually violent offenses perpetrated against adults) and sexual-offense-specific treatment, as operationalized by scales measuring three components of attitudes (i.e., affective, cognitive, and behavioral). Consistent with the extant literature (e.g., Malinen et al., 2014), we hypothesized that participants would demonstrate generally negative attitudes toward this population. Second, we sought to examine what sexual-offense-specific policies a young Canadian university sample would support—do they tend to support more incapacitation/control (IC)-based policies or more rehabilitative/reintegration (RR) based policies? In keeping with previous research suggesting that members of the public tend to endorse punitive policies for this group (e.g., Levenson et al., 2007) but may also support a combination of approaches (e.g., Brown et al., 2008), we hypothesized that participants would endorse policies of both types. Third, we sought to investigate how attitudes toward persons convicted of sexual offenses relate to policy endorsement. We hypothesized that people with more negative attitudes toward this group would show greater support for IC policy, per the limited research focusing on specific policy, such as community notification (Shackley et al., 2013). Conversely, we hypothesized that those with less punitive attitudes would show greater support for RR-based policy. Fourth, given that previous research has found demographic characteristics such as age, political orientation, gender, and education to be meaningfully associated with the valence of attitudes toward sex offending populations (e.g., Olver & Barlow, 2010), we sought to investigate to what extent participant demographics and attitudes may be uniquely associated with policy endorsement. We hypothesized that the associations projected under the third research aim would remain, however, given that this required the use of multivariate regression models, we did not advance specific predictions about unique associations of specific demographics to policy endorsement.

## Method

### A Word on Terminology

A movement is underway to replace the stigmatizing moniker “sex offender” in clinical and academic discourse with more person first terms that do not equate a person with an act or series of acts committed in the past (Seto, 2018). The measures used in the present study at the time of data collection (2015) preceded this widespread language shift, however, and make routine use of the term “sex offender.” Thus, this language and related terminology are used hereafter where necessary to accurately reflect the information presented to participants.

### Participants

Participants included 333 undergraduate students recruited from introductory psychology classes at a large medical doctoral university in Canada. In exchange for volunteering to complete the survey based on a brief description of the subject, participants received additional course credit (which they received even if they withdrew their participation). The majority of the sample self-identified as White (75.1%), 15% self-identified as Asian, and the remaining 10% self-identified from other Black, Indigenous, and Persons of Color (BIPOC) ethnoracial groups. The majority of the sample self-identified as female (81.2%). Most participants were between the ages of 18 and 24 years (91.3%), 4.5% were between 25 and 34 years, and the remaining 0.9% were between 35 and 44 years. With regard to education, 73.9% of the participants had completed some postsecondary studies, while 24.2% identified high school as their highest level of education. Most participants reported their intimate relationship status as single (91.7%) and reported having no children (96.7%).

### Materials

To achieve our four research aims, we administered well-established measures of attitudes, beliefs, and feelings toward people who commit sex crimes, and concordant general social psychology constructs (e.g., feeling thermometer [FT]), and a structured measure of sexual offense management and prevention policies developed for the purposes of the present study.

#### Attitudes Toward Treatment of Sex Offenders Scale

The Attitudes Toward Treatment of Sex Offenders (ATTSO) Scale is a self-report questionnaire developed to assess public attitudes toward treatment for people who have sexually offended (Wnuk et al., 2006). The ATTSO is completed using a 5-point Likert Scale ranging from “Strongly Disagree,” to “Undecided,” to “Strongly Agree”; to ensure consistency in interpretation across measures (i.e., the trend that higher scores reflect attitudes that are more positive), the valence of the original scale was reversed. In addition, item 13 (original item 25 “Sex offenders should be executed”) was not included, as Canada does apply capital punishment.

#### Attitudes Toward Sex Offenders and the Criminal Justice System Scale

The 25-item Attitudes Toward Sex Offenders and the Criminal Justice System (ATSOCJS) Scale was developed by Olver and Barlow (2010) to evaluate the attitudes toward people who have sexually offended and how they are managed by the criminal justice system in Canada. The scale comprises 21 items that are answered on a 5-point Likert-type scale (from Strongly agree to Strongly Disagree), and four open-ended items; this is a modification of the 3-point scale used in the original study and was undertaken to increase consistency with the other scales used in the study, and to provide more response options and variability. The open-ended items involve estimates regarding timespans and percentages (e.g., rates of recidivism) and were constructed to avoid influencing or biasing participant responses via constraints, anchors, or cues. Total possible scores on the 21 closed-ended items range from 21 to 105, with higher scores indicating more rehabilitative attitudes, and lower scores more negative and punitive attitudes.

#### Social Distance Scale

In an attempt to measure the behavioral component of attitudes toward people who have sexually offended, Willis and Colleagues (2013) developed a measure of social distance and anticipatory behavior related to this group, based on Social Distance Scales (SDS; Bogardus, 1925). This 8-item scale queries the extent to which participants would be willing to have a “sex offender” released from prison as a neighbor, colleague, boss, acquaintance, member of church/sports club/community group, close friend, partner in marriage/civil union, and son-in-law. Participants respond using a 5-point Likert scale (most definitely not to most definitely), with higher scores indicative of more positive attitudes and willingness to engage this group.

#### Feeling Thermometer

To gauge the emotional aspect of attitudes toward people who have sexually offended, as has been measured in previous research (e.g., Malinen et al., 2014), participants were asked to rate how they feel about them using an FT. Participants rated how they felt about released “sex offenders” on a scale from 0 (very negative) to 100 (very positive).

#### Sex Offender Policy Scale

The 28-item Sex Offender Policy (SOP) Scale was created for the current program of research, to identify which policies the Canadian public would be in favor of and which they would oppose. Items were derived from a literature review of existing control policies and reintegration strategies, completed by the first author. A review of evidence-based practices, such as the RNR approach to offender management and risk factors relevant to sexual recidivism was also completed, to identify potential policies that could be implemented in Canada. Both RR-based and punitive/control-based policies currently in practice were included as items in the survey. A multi-step review and revision process involving graduate students with specialized knowledge, laypersons, and subject matter experts was undertaken to populate the survey. Three content experts that the authors identified as well-established academic and clinical professionals in the field of sexual violence research were consulted, and endorsed the survey items in terms of comprehensiveness, content accuracy, and coverage. In effect, this study also represents an initial validation of the scale.

Results of a principal components analysis (PCA) with Varimax rotation on the present sample identified two components with 26 out of 28 items loading at 0.40 or higher. Approximately half of the items on the scale represent IC-based policies. These policies are those with a predominantly punitive/control focus or that lack research evidence suggesting they effectively reduce recidivism. Approximately half of the items on the scale represent RR policies (RRPs). These policies are those with a predominantly rehabilitative focus, that have research evidence suggesting they effectively reduce recidivism, or that are consistent with empirically supported principles of correctional rehabilitation. Originally scored on a 5-point Likert scale (ranging from Strongly Oppose (1) to Neutral (3), to Strongly in Favor (5)), individual items were re-coded on a binary scale to identify the percentage of participants who endorsed (i.e., responded somewhat in favor or strongly in favor of) any particular item on the SOP survey. That is, items which were originally rated 1, 2, or 3 (strongly oppose—neutral ratings) were recoded as a 0, and items which were scored either a 4 or 5 (somewhat and strongly in favor ratings) were coded as a 1.

### Procedure

Ethical approval for the research program was obtained from the Behavioral Research Ethics Board of the authors’ academic institution. The survey was created and administered using Qualtrics online survey software. Each administration began with a consent to participate introduction letter, which was followed by a demographic questionnaire, operational definition of a “sex offender,” and then the collection of attitude and policy measures. Participants were instructed to consider the following definition of “sex offender” when completing the study measures: “*Contact Sex Offender, Adult victim*: an offender who has been convicted of a contact sexual offense against a non-consenting adult (over the age of 18 years). Offenses may include: sexual assault (rape), aggravated sexual assault.” This category was selected to avoid variability driven by the heterogeneity inherent in the term “sex offender” (e.g., persons convicted of viewing child sexual exploitation material vs. those convicted of rape against adults) and because of its relevance to the sample in question (i.e., adult students). The individual measures (i.e., the ATTSO, ATSOCJS, SDS, FT, and the SOP) were then presented in counterbalanced order to prevent ordering effects, although demographic questions were always presented first.

### Data Analytic Plan

The data were screened for potential problems with missing values. Missing data among participants was minimal (<5%). The bulk of data analyses were conducted using listwise deletion of missing cases, with the exception of the PCA for the SOP scale, which used pairwise deletion. To address the first and second research objectives, we generated descriptive statistics at the total scale, subscale, and item level for each attitude measure as well as the SOP scale. With regard to the SOP scale in particular, we also recoded the items in a binary manner (endorsed vs. not endorsed) to examine the policies endorsed for each policy type (i.e., IC vs. RR). For the third research objective, scores from the policy questionnaire were entered into correlational analyses along with scores from the attitude measures. The attitudes measures and demographic variables were also intercorrelated to examine the trends that may have relevance to policy endorsement. Correlations between categorical (binary or ordinal) demographic variables and continuous attitude and policy variables are tantamount to mean group comparisons (i.e., point biserial correlations for binary variables) or standard Pearson correlations (i.e., for ordinal variables), and were conducted and reported in a common correlation matrix (Tables 2 and 4) for space considerations. Finally, to accomplish the fourth research objective, a set of multiple regression analyses were conducted to examine predictor–criterion relationships between attitude measures and RR- and IC-based policy endorsement controlling for sample demographics.

## Results

### Undergraduate Attitudes and Perceptions of Persons who Commit Adult Sexual Violence

Table 1 presents means and standard deviations for scale, subscale, and criterion variable scores for the four attitude measures; main trends are briefly reported. For ATTSO scores, participants’ responses scores fell close to the midpoint (i.e., 42) at the total scale and item level suggesting that attitudes toward sexual offense treatment and rehabilitation were neutral. At the subscale level, mean item ratings suggested a tendency to endorse that persons who commit acts of sexual violence should be eventually be released (incapacitation subscale), that treatment has the potential to reduce risk (treatment ineffectiveness subscale); participants were less inclined toward coerced or mandated treatment (mandated treatment subscale).

**Table 1.** Scale and Item Descriptive Statistics for Attitudes Toward Persons who have Sexually Offended.

Measure	*N*	Scale total			Item average			α
		*M*	SD	Range	*M*	SD	Range	
ATTSO								
Incapacitation subscale	330	24.9	5.0	8–35	3.6	0.72	1.1–5.0	.82
Treatment ineffectiveness subscale	327	13.8	2.3	4–20	3.5	0.58	1.0–5.0	.74
Mandated treatment subscale	327	4.9	2.1	3–15	1.6	0.69	1.0–5.0	.81
Total	319	43.6	6.6	19–60	3.1	0.47	1.4–4.3	.81
ATSOCJS								
Systems subscale	322	29.7	5.1	11–41	2.7	0.46	1.0–3.7	.76
Rehabilitation subscale	315	33.5	5.0	15–48	3.4	0.50	1.5–4.8	.76
Total	308	63.3	9.2	26–83	3.0	0.44	1.2–4.0	.86
Criterion variable items								
Overall sexual recidivism (%)	329	—	—	—	56.3	18.8	10.0–99.0	—
Sexual recidivism post-treatment (%)	328	—	—	—	38.1	19.1	0.0–90.0	—
Recidivism change (overall—treatment)	327	—	—	—	18.2	13.4	−35.0–99.0	—
Estimated prison term (years)	325	—	—	—	5.0	5.8	0.1–80.0	—
Ideal prison term (years)	316	—	—	—	10.2	9.0	0.1–77.5	—
Other measures								
SDS	331	15.5	6.2	8–40	1.9	0.77	1–5	.92
FT	330	15.4	19.3	0–100	—	—	—	—

*Note*. All multi-item attitude measures scored on a 5-point Likert scale (1–5) with 3 as the midpoint. Higher scores on all four measures reflect more positive evaluations of persons who have sexually offended.

ATTSO = Attitudes Toward Treatment of Sex Offenders; ATSOCJS = Attitudes Toward Sex Offenders and the Criminal Justice System; SDS = Social Distance Scales; FT = feeling thermometer.

For the ATSOCJS scale, the mean total score and item average fell at the midpoint (*M* = 63.3 and 3.0, respectively). The subscale scores suggested mildly negative leaning attitudes toward sentencing, release, and community management (systems subscale), but slightly more positive attitudes toward the treatment and reintegration prospects (rehabilitation subscale) of this population. Scrutiny of the four open-ended items on the ATSOCJS scale demonstrated similar averages to Olver and Barlow (2010). The sample estimated a 5-year sentence for men convicted of sexual assault contrasted with a mean preferred sentence length at roughly double at 10 years. The estimated sexual reoffense rate following treatment at 38% was significantly lower than that of untreated individuals, which was estimated overall at 56%; the difference of about 18 percentage points amounted to a large and statistically significant treatment effect (*d* = 0.95, *p* < .001), despite the estimates being substantially higher than the official reported recidivism rates (Harris & Hanson, 2004).

Finally, for the other measures, the mean SDS total score and item average fell below the midpoint of 24 to engage socially with this population (i.e., “most definitely not, to definitely not” range). In turn, the mean FT score (range 0 [very negative] to 100 [very positive]) of 15.4 indicated negative feelings on the part of this predominantly female student sample toward men who have committed sexual assault.

### Interrelation of Attitudinal and Demographic Factors

Table 2 shows that the four attitude measures, their total and subscale scores, were intercorrelated. The treatment-related subscales and the incapacitation/systems subscale of the ATTSO and ATSOCJS had particularly large intercorrelations, while desire for less social distance and more positive feelings (SDS and FT, respectively) had broadly small to moderate associations with these measures. The demographic variables had smaller and less consistent associations, with male sex, conservative leaning political orientation, and being White having associations with more negative attitudes (mandated treatment and systems) and feelings toward men who have committed sexual assault.

**Table 2.** Intercorrelations of Attitudes Measures with Sample Demographics.

Measure	ATTSO				ATSOCJS			FT	Social distance
	Incapacitation	Treat. ineff.	Mandated treatment	Total	Systems	Rehabilitation	Total		
ATTSO									
Treatment ineffectiveness	.60***								
Mandated treatment	−.13*	−.22***							
Total	.93***	.74***	.14*						
ATSOCJS									
Systems subscale	.60***	.47***	.06	.64***					
Rehabilitation subscale	.79***	.55***	−.07	.76****	.69***				
Total	.77***	.56***	.00	.77**	.92***	.92***			
Other measures									
FT	.11	.16**	.15**	.18**	.24***	.12*	.18**		
SDS	.47***	.37***	−.01	.48**	.50***	.46***	.52***	.30***	
Demographics									
Age	.05	.01	−.06	.03	.08	.07	.09	.14*	.07
Sex	−.06	.01	−.17**	−.09	−.17**	−.09	−.14*	−.13*	−.09
Race	−.09	.01	.14*	−.04	.12*	−.11*	.01	.18**	−.07
Education	.05	.00	−.06	.01	.07	.10	.09	.03	.01
Income	.05	−.05	.14	.06	.04	.11	.09	−.10	−.11
Single	−.05	−.02	.12*	−.01	−.08	−.07	−.08	−.03	−.04
Political	−.08	−.13*	.06	−.08	−.19**	−.07	−.14*	−.06	−.10

* *p* < .05, ** *p* < .01, ***p < .001.

ATTSO = Attitudes Toward Treatment of Sex Offenders; FT = feeling thermometer; ATSOCJS = Attitudes Toward Sex Offenders and the Criminal Justice System; CJS = Criminal Justice System; SDS = Social Distance Scales.

### SOP Endorsement

Table 3 includes individual item percent agreement values for specific policies comprising the SOP scale. When a binary total mean score was totaled for each component, an average of 10/14 RRP items were endorsed along with 7/10 ICP items. Participants endorsed approximately 70% of both RR and IC policies. The overall sample was generally in favor of the majority of the IC policies, when considering them individually. Most participants (93.4%) were in favor of all men committed of sexual offenses registering on a national sexual offender registry for 10 years post-release, 73.2% were in favor of lifelong registration, but only a small majority (53.2%) were in favor of making information on the registry public. A minority (31.5%) of the sample was in agreement that only high-risk individuals should have to serve prison time for their crimes. About two-thirds (66.8%) of the sample endorsed mandatory minimum sentences, while slightly under half (49.5%) felt that only contact sex offenses should have minimum mandatory sentences, or that released individuals should be placed on sex drive lowering medication (48.5%).

**Table 3.** SOP Scale Item Mean and Standard Deviations.

Item/total score	*N*	*M*	SD	% A
1. Sex offenders should have to be registered with the NSOR, for 10 years after they are released from prison.	333	4.5	0.71	93.4
2. Sex offenders should have to be registered with NSOR for life.	332	4.0	1.1	73.2
3. Information included on the NSOR should be made available to the public.	333	3.3	1.3	53.5
4. All sex offenders should have to serve time in prison for their crimes.	331	4.3	.93	84.6
5. Only high-risk sex offenders should have to serve time in prison for their crimes.	330	2.6	1.5	31.5
6. All sexual crimes should have minimum mandatory sentences.	331	3.8	1.2	66.8
7. Only sexually violent crimes (those involving direct physical contact with a victim) should have minimum mandatory sentences.	331	3.2	1.4	49.5
8. All high-risk sex offenders should be under an 810 peace bond after they have completed their sentence for a sexual crime.	331	4.1	1.1	77.3
9. All high-risk sex offenders should be made Long Term Offenders as part of their sentence.	330	4.2	.89	80.6
10. All high-risk sex offenders should be sentenced as DO.	333	3.7	1.1	64.3
11. Sex offenders with a high sex drive should have to take drug treatments to lower their sex drive when released from prison.	330	3.3	1.3	48.5
13. Sex offenders on probation or parole should also have to wear GPS tracking devices.	329	3.9	1.0	71.1
14. A sex-offender-specific therapy program should be offered to sex offenders in prison.	330	4.6	0.70	93.0
15. A sex offender-specific therapy program should be offered to sex offenders on probation or parole.	333	4.5	0.81	91.0
16. In order to keep up sex offenders’ therapy gains from prison, the CJS should offer therapy programs in the community for offenders who have finished their sentences.	330	4.5	0.80	90.6
17. Therapy related to personal relationship skills should be offered to sex offenders in prison.	331	4.3	0.84	86.7
18. Therapy related to personal relationship skills should be offered to sex offenders on probation or parole.	331	4.3	0.84	84.9
19. Criminal Justice programs to help sex offenders to find jobs once they return to the community should be offered to sex offenders in prison.	331	3.5	1.1	57.4
20. Criminal Justice programs to help sex offenders to find jobs in the community, after they are released from prison, should be offered to sex offenders on probation or parole.	330	3.7	1.1	64.5
21. Criminal Justice programs to help sex offenders find stable housing once they return to the community, should be offered to sex offenders in prison.	330	3.3	1.2	49.1
22. Criminal Justice programs to help sex offenders find stable housing should be offered to sex offenders on probation or parole.	330	3.5	1.1	55.8
23. Halfway houses only for sex offenders should be available in the community.	328	3.6	1.1	60.7
24. Volunteer options (e.g., to work a position in the kitchen or library) should be offered to sex offenders in prison.	333	3.6	1.1	59.2
25. Leisure/recreational options (e.g., ability to engage in sports/fitness, a book library) should be offered to sex offenders in prison.	332	3.7	1.1	63.6
26. COSA should be available for interested high-risk sex offenders across Canada.	331	4.2	0.91	83.1
28. There should be more Criminal Justice System support, beyond simple parole or probation resources, for sex offenders who request it, in the community.	330	4.0	0.96	74.2
Rehabilitation/reintegration policy total (14 items: 14–26, 28)	305	55.4	9.2	—
IC policy total (10 items: 1–4, 6, 8–11, 13)	317	39.1	5.8	—

*Note*. Items 12 and 27 are excluded as they are specific to child sexual victimization. %A = % Agree.

COSA = Circles of Support and Accountability; IC = Incapacitation/control; GPS = Global Positioning System; SOP = Sex Offender Policy; NSOR = National Sex Offender Registry; CJS = Criminal Justice System; DO = Dangerous Offender.

The majority of the sample was in favor of the majority of the RR policies as well. Over 90% of participants were in favor of providing a sexual-offense-specific therapy program both in and outside of prison, and that additional therapy programs should be offered to persons convicted of sex offenses upon release to the community. In all, 63.6% of participants were in favor of providing leisure/recreation programs in prison. Nearly half of the respondents (49.1%) were in favor of having institutional programs to help this population find stable housing in the community and 55.8% were in favor of offering such programs while on probation or parole.

### Attitudinal and Demographic Correlates of SOP Endorsement

Table 4 reports the associations between the attitudes measures with policy endorsement and sex offender sentencing and treatment. In short, more favorable attitudes and desire for less social distance were positively associated with RR policy and inversely associated with IC policy; increasingly negative feelings were also associated with IC policy endorsement. Three of the attitudes measures (ATTSO, ATSOCJS, and SDS) had significant and broadly moderate correlations with lower estimated rates of sexual recidivism (overall and after treatment) and inverse association for preferred prison term for men convicted of sexual assault. Both ATSOCJS subscales and the ATTSO treatment ineffectiveness subscale had small significant correlations with treatment effect. The policy subscales had opposite patterns of association with estimated recidivism and preferred prison term. RR policy endorsement was associated with lower estimated recidivism rates, positive treatment effect, and lower preferred prison term, while IC policy endorsement was associated with higher estimated recidivism rates, negative anticipated treatment response, and longer preferred prison terms. The demographic variables had weaker and less consistent associations; increasing age and ethnic minority status were associated with RR policy endorsement, whereas conservative political orientation and female sex were associated with IC policy endorsement.

**Table 4.** Associations between Attitudes and Demographics Measures With SOP and Criterion Variables.

Measure	SOP		Sexual recidivism rates			Ideal prison term
	RRP	ICP	Overall	Treated	Txt effect	
SOP						
RRP	—	−0.07	−0.12*	−0.20***	0.13*	−0.32***
ICP	−0.07	—	0.30***	0.37***	−0.12*	0.38***
ATTSO						
Incapacitation	0.50***	−0.44***	−0.38***	−0.43***	0.10	−0.48***
Treatment ineffectiveness	0.41***	−0.29***	−0.27***	−0.35***	0.14*	−0.44***
Mandated treatment	0.39***	−0.20***	−0.11*	−0.06	−0.06	0.13*
Total	0.40***	−0.50***	−0.42***	−0.47***	0.09	−0.48***
ATSOCJS						
Systems subscale	0.35***	−0.53***	−0.50***	−0.53***	0.08	−0.41***
Rehabilitation subscale	0.51***	−0.51***	−0.34***	−0.41***	0.14*	−0.48***
Total	0.48***	−0.57***	−0.45***	−0.52***	0.13*	−0.48***
Other measures						
FT	−0.10	−0.26***	−0.08	−0.15**	0.08	−0.08
SDS	0.32***	−0.31***	−0.26***	−0.31***	0.09	−0.24***
Demographics						
Age	0.13*	0.04	−0.01	−0.01	−0.02	−0.04
Sex	0.05	0.12*	0.13*	0.12*	0.01	0.05
Race	−0.18***	−0.10	−0.05	−0.10	0.06	−0.02
Education	0.07	−0.04	−0.03	−0.03	0.00	−0.05
Income	−0.05	0.01	−0.06	−0.02	−0.04	0.00
Single	−0.13	−0.03	v0.01	−0.06	0.06	0.06
Political	−0.04	0.14*	0.05	0.10	−0.06	0.15*

*** *p* < .001, **p < .01, *p < .05.

ATTSO = Attitudes Toward Treatment of Sex Offenders; ATSOCJS = Attitudes Toward Sex Offenders and the Criminal Justice System; ICP = Incapacitation/control policy; FT = feeling thermometer; SOP = Sex Offender Policy; RRP = rehabilitation/reintegration policy.

Table 5 reports the results of multiple regression analyses of attitudinal measures predicting policy endorsement controlling for demographic variables. Model 1 demonstrates that mandated treatment (ATTSO), negative feelings, and ethnic minority status were uniquely associated with lower RR policy endorsement while increasing age and rehabilitative supportive attitudes (ATSOCJS) were uniquely associated with stronger RR policy endorsement. Model 2, in turn, found that mandated treatment (ATTSO) and supportive systems and rehabilitation attitudes (both components of ATSOCJS), uniquely predicted decreased IC policy support, while conservative political orientation uniquely predicted increased IC policy support; negative feelings were also uniquely associated with weaker IC policy endorsement.

**Table 5.** Multiple Regression: Prediction of SOP Endorsement by Attitude Measures.

Regression model	*B*	SE	Β	*p*
Model 1	DV: Rehabilitation/reintegration policy (*N* = 270)			
ATTSO incapacitation	0.271	0.147	.154	.067
ATTSO treatment ineffectiveness	0.479	0.244	.122	.051
ATTSO mandated treatment	−1.34	0.212	−.309	**<.001**
ATSOCJS system	0.084	0.127	.048	.505
ATSOCJS rehabilitative	0.398	0.157	.222	**.012**
SDS	0.142	0.080	.101	.076
FT	−0.072	0.024	−.155	**.003**
Age	2.70	1.25	.103	**.032**
Race	−0.938	1.03	−.044	.362
(Constant)	26.40	4.26		
Model 1 results: *R* = .663, *R*^2^ = .440, *F*(269) = 22.66, *p* < .001				
Model 2	DV: ICP (*N* = 260)			
ATTSO incapacitation	−0.137	0.103	−.117	.187
ATTSO treatment ineffectiveness	0.016	0.173	.006	.925
ATTSO mandated treatment	−0.544	0.148	−.192	**<.001**
ATSOCJS system	−0.310	0.090	−.261	**.001**
ATSOCJS rehabilitative	−0.281	0.105	−.235	**.008**
SDS	0.027	0.057	.029	.636
FT	−0.040	0.016	−.133	**.013**
Sex	−0.344	0.767	−.023	.655
Political orientation	0.027	0.012	.112	**.027**
(Constant)	61.88	2.75		
Model 2 results: *R* = .624, *R*^2^ = .389, *F*(259) = 17.67, *p* < .001				

*Note*. Significant *p* values in bold font.

ATTSO = Attitudes Toward Treatment of Sex Offenders; ATSOCJS = Attitudes Toward Sex Offenders and the Criminal Justice System; ICP = Incapacitation/control policy; FT = feeling thermometer; SOP = Sex Offender Policy.

## Discussion

Perspectives of those impacted by sexual violence are important considerations in the development and implementation of related policy to manage and prevent sexual violence. That said, while public attitudes toward people with sexual offense histories have certainly influenced many policy decisions, it is not necessarily true that holding particular attitudes precludes the endorsement of particular policies. For instance, it is not currently known whether rejection of reintegrative approaches, or the exclusive endorsement of punitive approaches, follow directly from negative societal attitudes toward this group. Furthermore, because patterns observed among populations may not hold true, or might even be reversed, among subgroups (Kievit et al., 2013), replication and expansion of extant research studies are important endeavors. Thus, the current research sought to investigate, in a predominantly female Canadian university undergraduate sample, attitudes toward individuals convicted of crimes involving sexual violence and their treatment, and to identify sexual-offense-specific policies that Canadian students may support. The study also examined the associations between such attitudes and policy endorsement with this group. The overarching goal of this research is to inform effective public policy, and ultimately reduce sexual offending and associated harms both among university student populations and the broader public sphere.

Attitudes in this study were assessed broadly, using several scales tapping cognitive, affective, and behavioral dimensions. Contrary to expectations, the findings suggested that in many respects the sample’s overall attitudes could be described as neutral toward individuals who have sexually offended, as evidenced by mean scores near the midpoint on the ATTSO and ATSOCJS, and only slightly below the midpoint for the SDS scale. These results suggest that the sample does not hold unduly negative beliefs about this group, about engaging with them, or about sexual-offense-specific treatment. Variation was observed, however, among the attitudinal components measured. The affective component appeared most negative, with the mean score on the FT falling well below the midpoint. This finding is unsurprising given the stigma associated with the label *sex offender* (Evans & Cubellis, 2015), and also given that the affective component of attitudes toward people who have sexually offended has been found to be consistently negative, and to be the component least susceptible to change (Malinen et al., 2014; Willis et al., 2013). As mentioned briefly above, the SDS scale, which measures one’s willingness to engage with the target group, also fell below the midpoint. Specific items, such as that querying participants’ willingness to have “a sex offender released from prison as a close friend?” (item 6), revealed a reluctance to engage in close personal contact with this population.

The open-ended items on the ATSOCJS produced some notable findings. As observed in previous research, participants overestimated the rate of recidivism for adult contact sexual offense perpetrators (Brown et al., 2008, Olver & Barlow, 2010), with a mean estimate of approximately 56% that fell slightly below the estimated 60% rate of recidivism observed in Olver and Barlow’s (2010) original study of the ATSOCJS. That said, participants also estimated a significantly lower rate of recidivism for treated persons that amounted to a large treatment effect of nearly one full standard deviation. This finding suggests optimism, at least among a Canadian university student sample, that treatment can be helpful for this group.

### Policy Endorsement

As hypothesized, participants supported a mixture of policies. In fact, the sample was broadly in favor of a majority of policies falling under both the RR and the IC categories and endorsed similar proportions of the RR and IC policies presented to them. Consistent with existing research conducted elsewhere (Brown et al., 2008; Mears et al., 2008; Willis et al., 2010), these findings suggest that when provided with both punitive and rehabilitative policy options, members of the Canadian public in a university setting support a combination of measures to manage people with sexual offense histories as they attempt to reintegrate into the community. In our view, the support for diverse strategies is also entirely compatible with recent movements toward wide-ranging and multifaceted postsecondary sexual violence initiatives (McMahon et al., 2019; Richards, 2019). Of additional significant interest is the finding that endorsement of RR policy was not associated with IC policy endorsement. In other words, one’s endorsement of IC policies was essentially unrelated to his or her endorsement of RR policies, suggesting that policy endorsement is not unidimensional.

Specific IC policies supported by the participants included: the long-term use of registries, universal incarceration, GPS tracking, and mandatory minimum sentences. These results are in line with the findings from outside Canada, indicating that punitive approaches are widely supported (e.g., Anderson & Sample, 2008; Levenson et al., 2007; Schiavone & Jeglic, 2009). Despite their apparently popularity, evidence suggests that some of these policies have no impact on recidivism (e.g., Levenson & D’Amora, 2007; Nunes et al., 2007; Tewksbury & Jennings, 2010; Vasquez et al., 2008), and researchers have argued that such policies can overburden the justice system and negatively impact reintegration, thereby increasing the risk of reoffending (Harris et al., 2010; Schiavone & Jeglic, 2009). Thus, insofar as public support guides policy decisions, these findings suggest a potential area of concern for those interested in using policy to improve public safety.

Supported RR policies included the following: sexual-offense-specific treatment, housing assistance, COSA, and generally providing additional community support post-release. These results are encouraging because such programs have empirical support for their effectiveness in reducing sexual offending (Gannon et al., 2019; Hanson et al., 2009; Wilson et al., 2005).

### Associations among Attitude Measures and Policy Endorsement

As hypothesized, attitudes as measured by the four scales were associated with policy endorsement. Specifically, more positive attitudes, as measured by the cognitive and behavioral scales (ATTSO, ATSOCJS, and SDS), were significantly and positively associated with greater RR policy endorsement, and significantly negatively associated with greater IC policy endorsement. These findings are consistent with limited previous research, such as findings indicating that those with more negative attitudes were supportive of community notification (Shackley et al., 2013), which would be considered an IC policy. Interestingly though, the findings suggested an inverse relationship between supportive sentiments toward people who have sexually offended and endorsement of RR policies, or conversely, a positive relationship between negative feelings toward this group and greater endorsement of RR policies.

Multiple regression analyses further suggested that attitudes account for substantial variation in the endorsement of both policy types. More positive beliefs, more willingness to engage with, and more negative feelings toward this group were each uniquely associated with increased RR policy endorsement, consistent with the correlational analyses. The latter association is perhaps the least intuitive, as one might reasonably expect that persons with more negative feelings toward people who have sexually offended would be less inclined to endorse rehabilitative or reintegration-focused strategies. While the current data cannot speak to the cause of this phenomenon, one potential explanation is that those who feel most negatively toward this group are simply more inclined to endorse *any* type of policy that could offer some perceived protection from them. With regard to IC policies, more negative attitudes toward people who have sexually offended, and particularly negative beliefs and feelings, were uniquely associated with greater endorsement; this result is in line with previous research suggesting that those with more negative attitudes were supportive of restrictive policies (Shackley et al., 2013).

### Strengths, Limitations, and Future Directions

With respect to diversity, this study relied on a relatively homogeneous undergraduate student sample, consisting largely of young, single, and educated White females. Opportunities to explore personal and demographic characteristics as moderators were limited, which may limit the generalizability of certain findings from the current a sample to the broader population of North American university students, bearing in mind that university students, particularly young women, are uniquely impacted by sexual violence. Nonetheless, some of the results are likely of broader public interest. For instance, the findings that negative attitudes did not necessarily preclude endorsement of RR policies, and that RR and IC policy endorsement were effectively unrelated, both point to notable phenomena regardless of sample characteristics.

With regard to future research directions, this study can provide a model for further study utilizing broader sampling methods. In particular, replications would be valuable among other postsecondary settings, or among representative samples from Canada or other locales in which effective policy requires public engagement. Replication among more diverse samples could also allow for more complex analyses, including investigations of how identity factors (i.e., race, gender, etc.) might shed light on support for particular policies. For instance, BIPOC ethnoracial groups, including Black, Hispanic, and Indigenous persons, are highly overrepresented in prisons and jails in North America (Public Safety Canada, 2018; Travis et al., 2014) and it is possible that perpetrator ethnoracial status and/or gender could moderate associations with, and endorsements of, sexual violence prevention policy.

Given the heterogeneity inherent in sexual violence, research accounting for the potentially moderating influences of diversity in offending would also be of value (e.g., persons with histories of contact vs. non-contact offenses). Further research may also seek to survey policymakers with regard to whether awareness of such nuanced public perspectives might influence their decision-making.

## Conclusions

Knowledge of public attitudes and support for various policies have important practical implications for successful management of persons convicted of sexual crimes and their positive reintegration into the community (Harper & Hogue, 2015). The current findings provide useful preliminary information about Canadian university undergraduate attitudes toward this group and their support for various sexual-offending-related policies, as well as interesting insights into the nuanced relationships among the two; what are the implications? As Sample and colleagues (2011) have argued, public policy can serve symbolic functions, such as appeasing public concern, while also serving instrumental functions, such as influencing behavior. The current results suggest that even in contexts in which feelings toward people who have sexually offended are negative, options available to policymakers wishing to appease the public may not be limited to punitive and reactionary measures. It is incumbent upon researchers, clinicians, and policymakers alike, to continue exploring opportunities to implement effective and evidence-based approaches to reduce sexual violence.
